# Podocalyxin is crucial for the growth of oral squamous cell carcinoma cell line HSC-2

**DOI:** 10.1016/j.bbrep.2018.07.008

**Published:** 2018-08-07

**Authors:** Shunsuke Itai, Shinji Yamada, Mika K. Kaneko, Masato Sano, Takuro Nakamura, Miyuki Yanaka, Saori Handa, Kayo Hisamatsu, Yoshimi Nakamura, Yoshikazu Furusawa, Masato Fukui, Tomokazu Ohishi, Manabu Kawada, Hiroyuki Harada, Yukinari Kato

**Affiliations:** a Department of Antibody Drug Development, Tohoku University Graduate School of Medicine, 2-1 Seiryo-machi, Aoba-ku, Sendai, Miyagi 980-8575, Japan; b Department of Oral and Maxillofacial Surgery, Graduate School of Medical and Dental Sciences, Tokyo Medical and Dental University, 1-5-45, Yushima, Bunkyo-ku, Tokyo 113-8510, Japan; c New Industry Creation Hatchery Center, Tohoku University, 2-1, Seiryo-machi, Aoba-ku, Sendai, Miyagi 980-8575, Japan; d ZENOAQ RESOURCE CO., LTD., 1-1 Tairanoue, Sasagawa, Asaka-machi, Koriyama, Fukushima 963-0196, Japan; e Institute of Microbial Chemistry (BIKAKEN), Numazu, Microbial Chemistry Research Foundation, 18-24 Miyamoto, Numazu, Shizuoka 410-0301, Japan

**Keywords:** OSCC, oral squamous cell carcinoma, mAb, monoclonal antibody, Oral squamous cell carcinoma, OSCC, HSC-2, Podocalyxin, PODXL, Monoclonal antibody

## Abstract

Oral cancers constitute approximately 2% of all cancers, with the most common histological type being oral squamous cell carcinoma (OSCC), representing 90% of oral cancers. Although diagnostic technologies and therapeutic techniques have progressed, the survival rate of patients with OSCC is still 60%, whereas the incidence rate has increased. Podocalyxin (PODXL) is a highly glycosylated type I transmembrane protein that is detected in normal tissues such as heart, breast, and pancreas as well as in many cancers, including lung, renal, breast, colorectal, and oral cancers. This glycoprotein is associated with the progression, metastasis, and poor outcomes of oral cancers. PODXL overexpression was strongly detected using our previously established anti-PODXL monoclonal antibody (mAb), PcMab-47, and its mouse IgG_2a_-type, 47-mG_2a_. In previous studies, we also generated PODXL-knock out (PODXL-KO) cell lines using SAS OSCC cell lines, in order to investigate the function of PODXL in the proliferation of oral cancer cells. The growth of SAS/PODXL-KO cell lines was observed to be lower than that of parental SAS cells. For this study, PODXL-KO OSCC cell lines were generated using HSC-2 cells, and the role of PODXL in the growth of OSCC cell lines *in vitro* was assessed. Decreased growth was observed for HSC-2/PODXL-KO cells compared with HSC-2 parental cells. The influence of PODXL on tumor growth of OSCC was also investigated *in vivo*, and both the tumor volume and the tumor weight were observed to be significantly lower for HSC-2/PODXL-KO than that for HSC-2 parental cells. These results, taken together, indicate that PODXL plays an important role in tumor growth, both *in vitro* and *in vivo*.

## Highlights

• PODXL-knock out cell lines were established using HSC-2.
• The growth of HSC-2/PODXL-KO cells were lower than that of parental HSC-2.
• Tumor volumes of HSC-2/PODXL-KO cells were lower than that of HSC-2.
• PODXL has an important role in tumor growth *in vitro* and *in vivo*.

## 1 Introduction

Globally, oral cancers constitute approximately 2% of all cancers [1]. They can be histologically classified into the following types: squamous cell carcinoma, adenoid carcinoma, adenoid cystic carcinoma, mucoepidermoid carcinoma, and osteosarcoma. Approximately 90% of oral cancer burden is borne by oral squamous cell carcinoma (OSCC) [2]. Although diagnostic technologies and therapeutic techniques have progressed by leaps and bounds in recent decades, the survival rate of patients with OSCC has not improved. Consequently, the 5-year survival rate of patients with OSCC is still 60% [3]; however, the incidence rate of OSCC is increasing [4], [5].

Podocalyxin (PODXL) is a CD34-related highly glycosylated type I transmembrane protein [6], [7], [8], [9]. PODXL can be detected in normal tissues such as heart, breast, and pancreas [10] as well as in cancers including lung, renal, breast, colorectal, and oral cancers [9], [11], [12], [13], [14], [15]. PODXL overexpression is associated with the progression, metastasis, and poor outcomes of several cancer types [16], [17], [18], [19].

In our previous study, we established specific and sensitive anti-PODXL mAbs: PcMab-47 (mouse IgG_1_, kappa) [20], 47-mG_2a_ (mouse IgG_2a_-type of PcMab-47), and 47-mG_2a_-f (core fucose-deficient type of 47-mG_2a_) [21]. We demonstrated that 47-mG_2a_-f significantly reduced tumor growth in OSCC xenograft models. [21]. To investigate the function of PODXL in the growth of oral cancer cells, we previously generated PODXL-knock out (PODXL-KO) cell lines using SAS OSCC cell lines. The growth of SAS/PODXL-KO cell lines (BINDS-01) was observed to be lower than that of parental SAS cells. In this study, we further established PODXL-KO OSCC cell lines using HSC-2 (oral squamous cell carcinoma from oral cavity) cells and investigated the role of PODXL in the growth of OSCC cell lines both *in vitro* and *in vivo*.

## 2 Materials and methods

### 2.1 Cell lines

HSC-2 cells were obtained from Japanese Collection of Research Bioresources Cell Bank (Osaka, Japan). HSC-2/PODXL-KO cells (BINDS-02) were produced using CRISPR/Cas9 plasmids (Target ID: HS0000056763), targeting human PODXL (Sigma-Aldrich Corp., St. Louis, MO, USA). Three clones were selected for this study: HSC-2/PODXL-KO #1, HSC-2/PODXL-KO #2, and HSC-2/PODXL-KO #3. Parental HSC-2 and HSC-2/PODXL-KO cells were cultured in Dulbecco's Modified Eagle's Medium (DMEM; Nacalai Tesque, Inc., Kyoto, Japan), which was supplemented with 10% heat-inactivated fetal bovine serum (Thermo Fisher Scientific Inc., Waltham, MA, USA), 100 units/mL penicillin, 100 μg/mL streptomycin, and 25 μg/mL amphotericin B (Nacalai Tesque, Inc.), at 37 °C in a humidified atmosphere containing 5% CO_2_ and 95% air. BINDS-01 and BINDS-02 can be obtained from Kato's lab in Tohoku University Graduate School of Medicine (http://www.med-tohoku-antibody.com/topics/001_paper_cell.htm).

### 2.2 Flow cytometry

HSC-2/PODXL-KO cell lines were harvested following a brief exposure to 0.25% trypsin in 1 mM ethylenediaminetetraacetic acid (Nacalai Tesque, Inc.). After washing with 0.1% bovine serum albumin in phosphate-buffered saline (Nacalai Tesque, Inc.), cells were treated with primary mAbs for 30 min at 4 °C, followed by treatment with Alexa Fluor 488-conjugated anti-mouse IgG (1:2000; Cell Signaling Technology, Danvers, MA, USA). Fluorescence data were obtained using SA3800 Cell Analyzer (Sony Corp., Tokyo, Japan).

### 2.3 In vitro proliferation assay

*In vitro* cell proliferation was measured using Cell Cloning Kit-8 (CCK-8; Dojindo, Kumamoto, Japan), which can assess cell viability. Cells were plated (1500, 3000, and 6000 cells/100 μL/well) in quintuple wells in 96-well plates and were incubated for 48 h. After adding 10 μL of CCK-8 to each well, the plates were incubated for 4 h at 37 °C. Subsequently, the absorbance was recorded at 450 nm using iMark microplate reader (Bio-Rad Laboratories, Inc., Berkeley, CA). The mean absorbance of the 5-well set was obtained at 48 h after cell seeding. All data were expressed as the mean ± SEM. Statistical significance was analyzed using Tukey–Kramer's test. *P*-values < 0.05 were considered statistically significant.

### 2.4 In vivo proliferation assay

Five-week-old, female BALB/c nude mice were purchased from CLEA Japan (Tokyo, Japan). Seven-week-old mice were used for the *in vivo* proliferation assay. Cells (0.1 mL of 5 × 10^7^ /mL in DMEM) were mixed with 0.1 mL of BD Matrigel Matrix Growth Factor Reduced (BD Biosciences, San Jose, CA, USA). A 200 μL suspension (containing 5 × 10^6^ cells) was subcutaneously injected into the left flanks of nude mice. In this experiment, the following four cell lines were used: HSC-2, HSC-2/PODXL-KO #1, HSC-2/PODXL-KO #2, and HSC-2/PODXL-KO #3. Each group included eight mice. The tumor diameter was measured at day 7, 14, and 21 after injecting cancer cell lines using calipers, and the tumor volume was calculated using the following formula: volume = W^2^ × L/2, where W is the short diameter, and L is the long diameter. All mice were euthanized 21 days after cell implantation, and the tumor weight was measured. All data were expressed as mean ± SEM. Statistical analysis was performed using Tukey–Kramer's test. *P*-values < 0.05 were considered statistically significant.

### 2.5 Hematoxylin and eosin staining

After euthanizing mice at day 21, histopathological specimen sections (thickness, 4 µm) were deparaffinized in xylene, rehydrated, and stained with hematoxylin and eosin.

## 3 Results and discussion

**Fig. 1:**
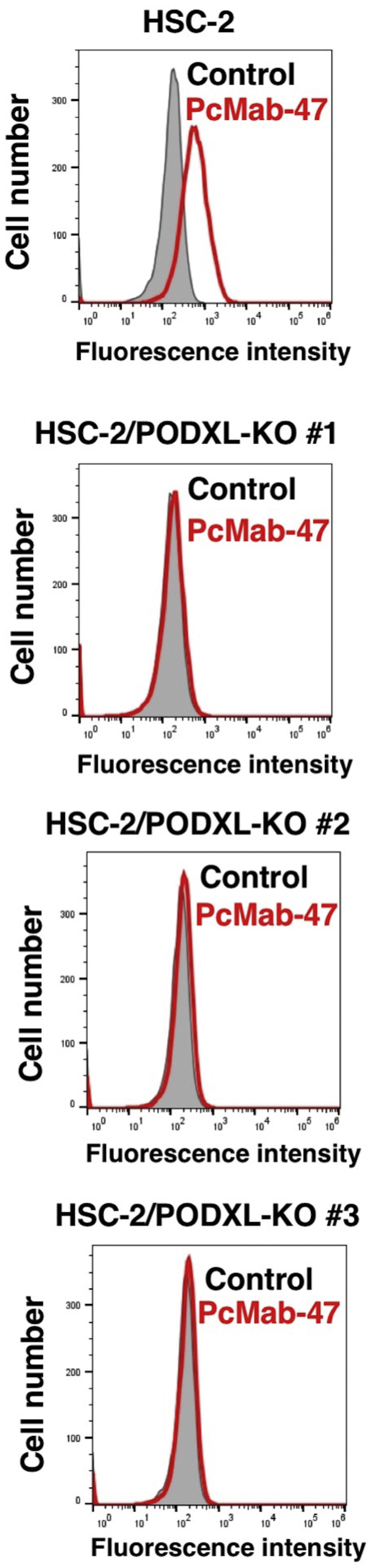
Flow cytometry using HSC-2/PODXL-KO cell lines. HSC-2, HSC-2/PODXL-KO #1, HSC-2/PODXL-KO #2, and HSC-2/PODXL-KO #3 were treated with PcMab-47 (10 μg/mL), followed by secondary antibodies. Gray peak, negative control Red peak, PcMab-47.

For this study, CRISPR/Cas9 plasmids targeting human PODXL were used to generate three PODXL-KO OSCC cell lines: HSC-2/PODXL-KO #1, #2, and #3. Before initiating the study, PODXL expression was confirmed in these cell lines. As shown in Fig. 1, anti-human PODXL mAb, PcMab-47 reacted with the HSC-2 parental cell line, but did not react with any of the three HSC-2/PODXL-KO cell lines.

**Fig. 2:**
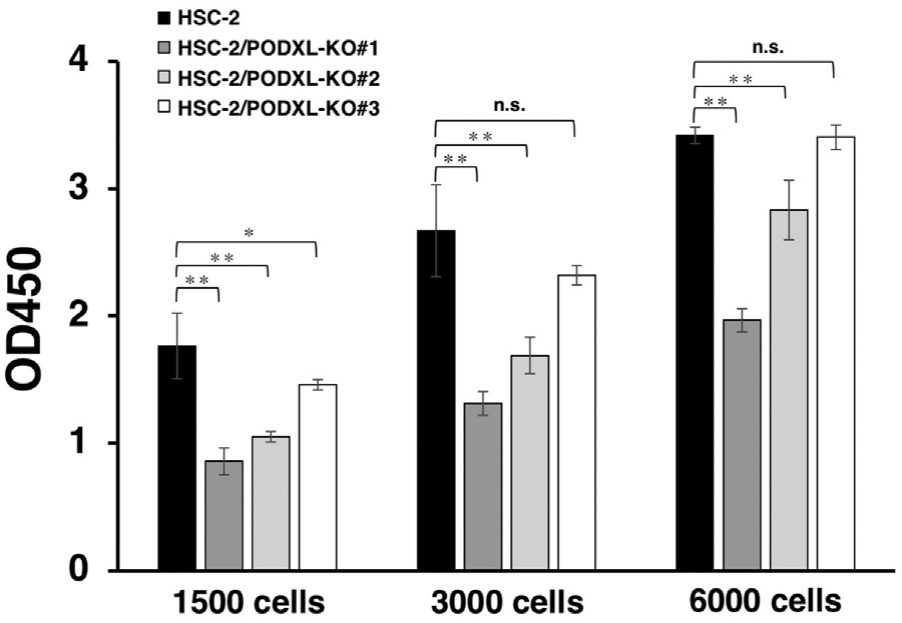
*In vitro* functional analysis of PODXL using PODXL-KO OSCC lines. HSC-2, HSC-2/PODXL-KO #1**,** HSC-2/PODXL-KO #2, and HSC-2/PODXL-KO #3 cell lines were plated (1500, 3000, and 6000 cells/100 μL/well) in quintuple wells in 96-well plates and were incubated for 48 h. After adding 10 μL of CCK-8 to each well, plates were incubated for 4 h at 37 °C. The values are mean ± SEM. The absorbance was recorded at 450 nm (*P < 0.05, **P < 0.01, Tukey–Kramer's test).

Next, we investigated the potential involvement of PODXL in the stimulation of *in vitro* OSCC cell growth. At every plated cell density (1500, 3000, and 6000 cells/well), the growth of HSC-2/PODXL-KO #1 and #2 cells was significantly lower than that of HSC-2 cells (Fig. 2). In contrast, HSC-2/PODXL-KO #3 showed significantly less growth than HSC-2 cells only when at a plated cell density of 1500 cells/well. These results indicate that while PODXL is an important factor for *in vitro* HSC-2 cell growth, many other factors may be involved in the induction or inhibition of HSC-2 cell growth.

**Fig. 3:**
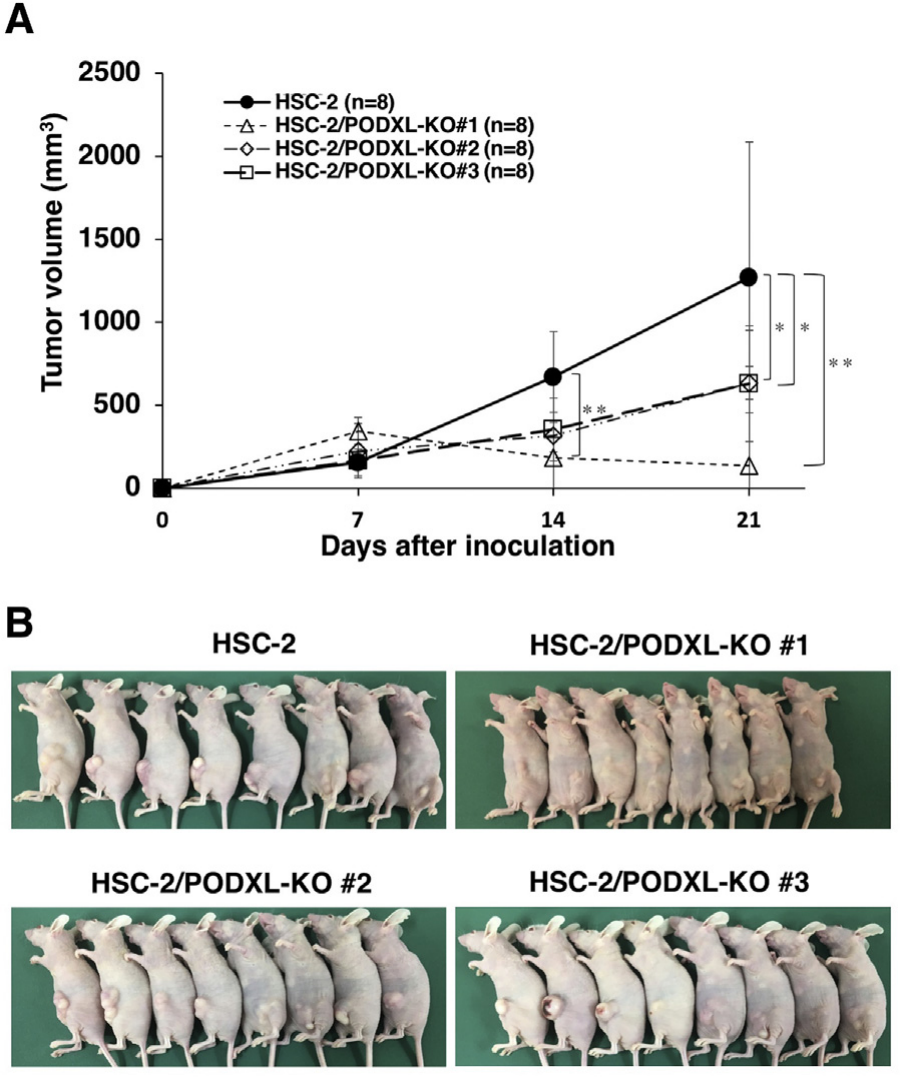
*In vivo* functional analysis of PODXL using PODXL-KO OSCC lines. HSC-2, HSC-2/PODXL-KO #1, HSC-2/PODXL-KO #2, and HSC-2/PODXL-KO #3 cell lines were injected subcutaneously into female BALB/c mice. (A) The tumor volume was measured at day 7, 14, and 21 after inoculation. The values are presented as mean ± SEM. Asterisks indicate statistical significance between HSC-2 and HSC-2/PODXL-KO cell lines (*P < 0.05, **P < 0.01, Tukey–Kramer's test). (B) Mice were euthanized at day 21 and their photos were taken at the same day.

The role of PODXL in OSCC tumor growth *in vivo* was further examined by comparing the growth of four cell lines, parental HSC-2, HSC-2/PODXL-KO #1, HSC-2/PODXL-KO #2, and HSC-2/PODXL-KO #3, which were transplanted subcutaneously into nude mice. On days 7, 14, and 21 after inoculation, tumor volumes of them were measured (Fig. 3). On day 7, no difference in tumor volumes were observed between parental HSC-2 and HSC-2/PODXL-KO cell lines. The tumor volume of HSC-2/PODXL-KO #1 cell lines was significantly lower than that of parental HSC-2 cell lines on day 14, although the tumor volumes for HSC-2/PODXL-KO #2 and #3 cell lines were similar to those arising from the parental cell line, HSC-2 (Fig. 3A). On day 21, tumor volumes arising from the transplantation of HSC-2/PODXL-KO #1, #2, and #3 cell lines were significantly lower than those arising from the parental HSC-2 cell lines (Fig. 3A). Subcutaneous tumors arising from parental HSC-2, HSC-2/PODXL-KO #1, HSC-2/PODXL-KO #2, and HSC-2/PODXL-KO #3 on day 21 are shown in Fig. 3B. *In vivo* analysis revealed that HSC-2/PODXL-KO #1 cell lines resulted in the smallest tumor growth among the three HSC-2/PODXL-KO cell lines (Fig. 3A), which is consistent with the *in vitro* result (Fig. 2).

**Fig. 4:**
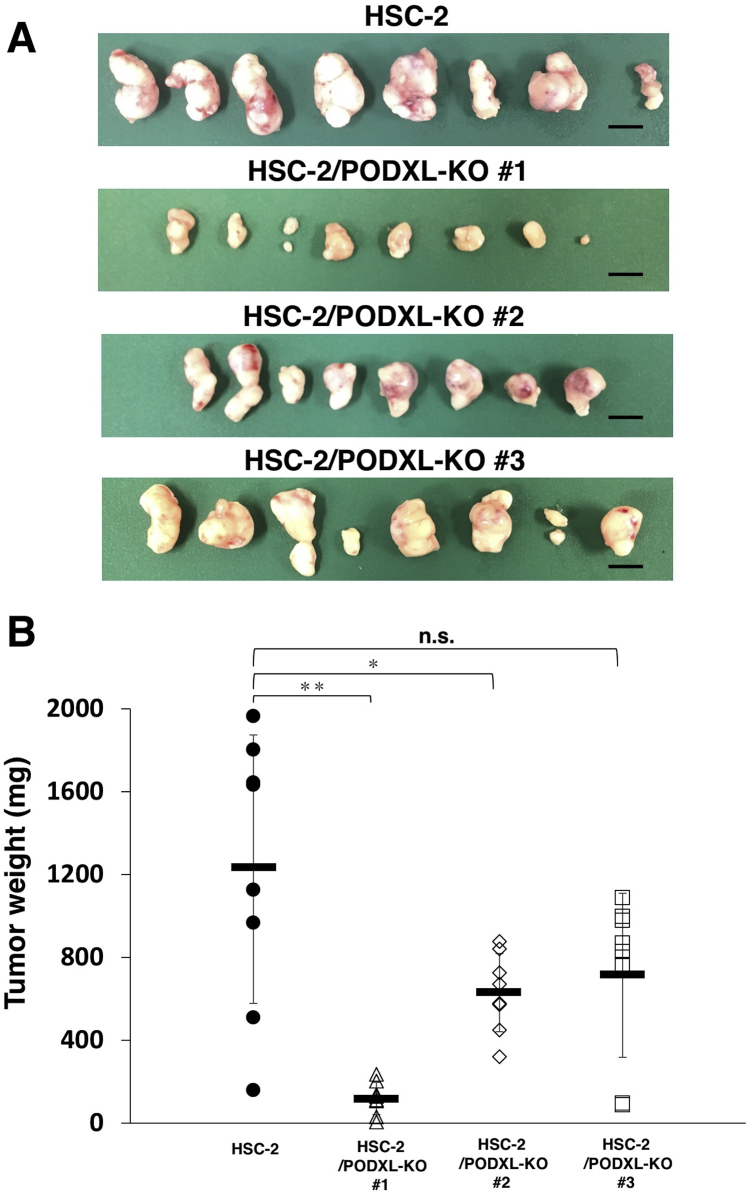
Comparison of the tumor size in the *in vivo* functional analysis of PODXL using PODXL-KO OSCC lines. (A) After euthanizing mice at day 21, tumors were resected. Scale bar = 1 cm. (B) Tumor weights were measured after tumor resection on day 21. The values are presented as mean ± SEM. Asterisks indicate statistical significance between HSC-2 and HSC-2/PODXL-KO cell lines (*P < 0.05, **P < 0.01, Tukey–Kramer's test).

**Fig. S1:**
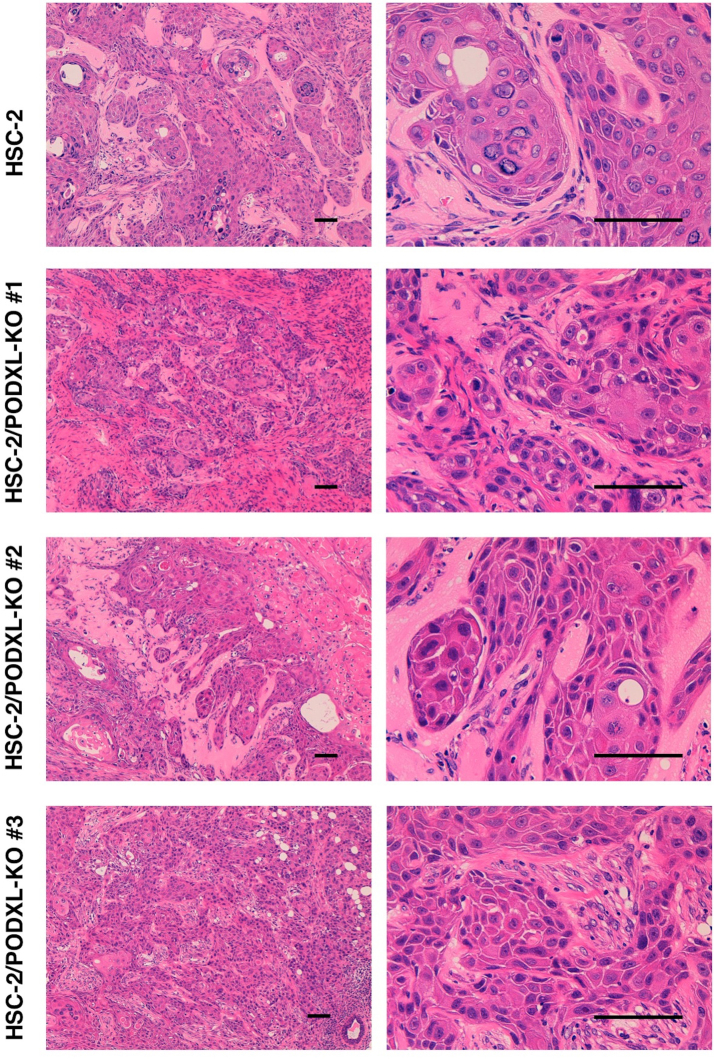
Supplementary Figure 1. Hematoxylin and eosin staining of the tumor tissue. Histopathological sections (4 µm thick) were deparaffinized in xylene, rehydrated, and stained using hematoxylin and eosin. Scale bar = 100 µm.

On day 21 after inoculation, all tumors were measured, following the resection of mice (Fig. 4A). As depicted in Fig. 4B, the tumor weight from HSC-2/PODXL-KO #1 and #2 cell lines were significantly lower than those from parental HSC-2 cell lines. The tumor weight from HSC-2/PODXL-KO #3 was not significantly different from that of parental HSC-2 cell line (Fig. 4B), whereas the tumor volumes from HSC-2/PODXL-KO #3 cell lines were significantly lower than those from parental HSC-2 cell lines (Fig. 3A). Hematoxylin and eosin staining of resected tumors of parental HSC-2, HSC-2/PODXL-KO #1, HSC-2/PODXL-KO #2, and HSC-2/PODXL-KO #3 on day 21 are shown in Supplementary Fig. 1.

Taken together, our results indicate that PODXL plays an important role in the growth of tumor in oral cancers. The HSC-2/PODXL-KO cell lines, which were generated in this study, are promising tools that can be used in future studies to elucidate the function of PODXL in the proliferation of oral cancer.

## Funding

This research was supported in part by AMED under Grant Numbers: JP18am0101078 (Y.K.), JP18am0301010 (Y.K.), and JP18ae0101028 (Y.K.), and by JSPS KAKENHI Grant Number 17K07299 (M.K.K.) and Grant Number 16K10748 (Y.K.).
